# Impact of pretreatment and downstream processing technologies on economics and energy in cellulosic ethanol production

**DOI:** 10.1186/1754-6834-4-27

**Published:** 2011-09-05

**Authors:** Deepak Kumar, Ganti S Murthy

**Affiliations:** 1 Biological and Ecological Engineering, Oregon State University, Corvallis, USA

**Keywords:** Grass straw, cellulosic ethanol, pretreatment, process model, process economics.

## Abstract

**Background:**

While advantages of biofuel have been widely reported, studies also highlight the challenges in large scale production of biofuel. Cost of ethanol and process energy use in cellulosic ethanol plants are dependent on technologies used for conversion of feedstock. Process modeling can aid in identifying techno-economic bottlenecks in a production process. A comprehensive techno-economic analysis was performed for conversion of cellulosic feedstock to ethanol using some of the common pretreatment technologies: dilute acid, dilute alkali, hot water and steam explosion. Detailed process models incorporating feedstock handling, pretreatment, simultaneous saccharification and co-fermentation, ethanol recovery and downstream processing were developed using SuperPro Designer. Tall Fescue (*Festuca arundinacea Schreb*) was used as a model feedstock.

**Results:**

Projected ethanol yields were 252.62, 255.80, 255.27 and 230.23 L/dry metric ton biomass for conversion process using dilute acid, dilute alkali, hot water and steam explosion pretreatment technologies respectively. Price of feedstock and cellulose enzymes were assumed as $50/metric ton and 0.517/kg broth (10% protein in broth, 600 FPU/g protein) respectively. Capital cost of ethanol plants processing 250,000 metric tons of feedstock/year was $1.92, $1.73, $1.72 and $1.70/L ethanol for process using dilute acid, dilute alkali, hot water and steam explosion pretreatment respectively. Ethanol production cost of $0.83, $0.88, $0.81 and $0.85/L ethanol was estimated for production process using dilute acid, dilute alkali, hot water and steam explosion pretreatment respectively. Water use in the production process using dilute acid, dilute alkali, hot water and steam explosion pretreatment was estimated 5.96, 6.07, 5.84 and 4.36 kg/L ethanol respectively.

**Conclusions:**

Ethanol price and energy use were highly dependent on process conditions used in the ethanol production plant. Potential for significant ethanol cost reductions exist in increasing pentose fermentation efficiency and reducing biomass and enzyme costs. The results demonstrated the importance of addressing the tradeoffs in capital costs, pretreatment and downstream processing technologies.

## Background

Bioethanol, a renewable energy source, is one of the alternatives to petroleum. Over last decade, bioethanol production has increased from 6.2 (year 2000) to 50 billion liters/year (year 2010) in United States. Number of ethanol production plants have increased from 54 (year 2000) to 189 (year 2010) [1]. Most of this growth in ethanol has been from first generation corn ethanol. Ethanol can be used as transportation fuel in existing gasoline vehicles after blending with gasoline, such as E10 - a mixture of 10% ethanol and 90% of gasoline by volume. Presently most of the ethanol is produced from fermentation of sugars in feedstocks such as sugarcane, sweet sorghum, corn, cassava, wheat and constitute what are known as first generation biofuel [2]. However, challenges such as capacity limitations (feedstock availability and supply), food vs. fuel issues, high feedstock prices, land and fresh water use, intensive agricultural inputs have led to investigation of second generation biofuels that address some of these concerns. Lignocellulosic biomass (e.g. agricultural residues, forestry wastes, grasses, wastepaper, municipal wastes and various industrial wastes), due to their abundance and low cost, are potential alternatives to serve as feedstock for the second generation ethanol production [3-5].

**Figure 1:**
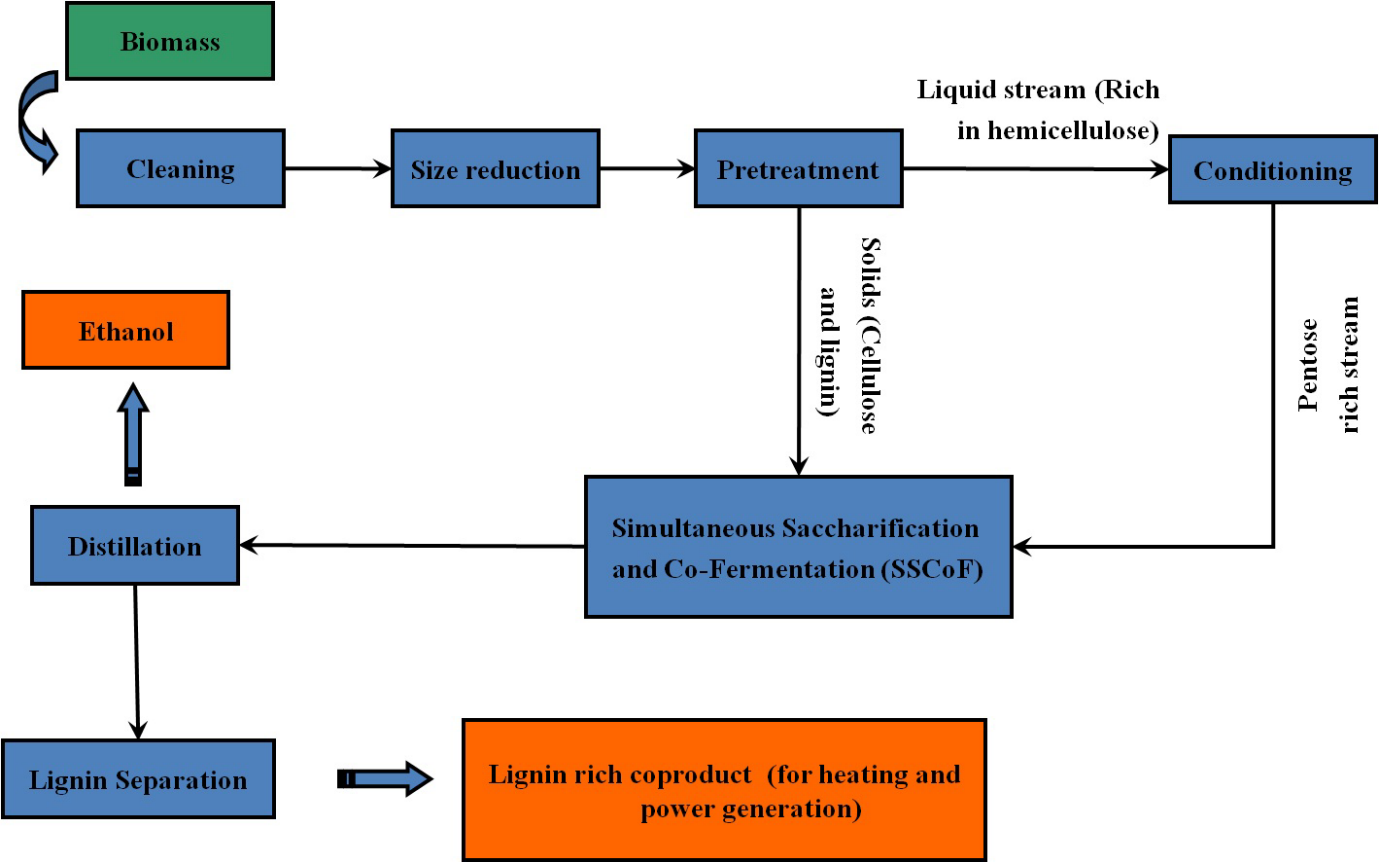
**Generic Cellulosic Ethanol Production Process**. Figure illustrates the common unit operations in ethanol production plants. Separation of liquid and solid after pretreatment is not necessary for all pretreatment technologies.

Lignocellulosic feedstocks are composed of mainly cellulose, hemicellulose, lignin, extractives and ash consisting of inorganic minerals. Production of cellulosic ethanol via biological conversion consists of three critical steps: pretreatment of biomass, hydrolysis of sugar polymers (cellulose, hemicellulose etc.) to sugar monomers and fermentation of sugar monomers to ethanol. A generic cellulosic ethanol production process is shown in Figure 1. Hydrolysis of sugar polymers can be achieved chemically by using acid or biologically using enzymes. Enzymatic hydrolysis is favored over acid hydrolysis due to lower energy consumption (natural gas, electricity), mild operating conditions, high sugar yields, and lower capital and maintenance cost of equipment [6,7]. However, in the case of lignocellulosic biomass, recalcitrant and heterogeneous structure of the biomass poses a fundamental challenge to depolymerization of cellulose during the enzymatic hydrolysis process. Enzyme accessibility is restricted by the lignin and hemicellulose and enzymes tend to irreversibly bind to lignin which slows down the process [8].

Several pretreatment methods, aimed at enhancing the susceptibility of lignocellulosic biomass to enzymes, have been investigated by researchers for degradation of hemicellulose and lignin and to break the crystalline structure of cellulose. Pretreatment techniques are mainly classified as: physical (e.g. mechanical comminution), chemical (e.g. dilute acid or alkali, Ammonia percolation), physio-chemical (e.g. steam explosion, Ammonia fiber expansion (AFEX)), and biological pretreatments (e.g. using white rot fungi) [2,9,10]. All of these techniques have been investigated by many researchers for different feedstocks [11-17]. Due to lack of commercial scale biochemical cellulosic ethanol facilities, most of these technologies have only been tested on laboratory/pilot scale. Four different pretreatment methods: dilute acid, dilute alkali, hot water and steam explosion, modeled in this study are among the most commonly used pretreatment methods [2,3,9,10,18]. Dilute acid pretreatment is one of the extensively investigated method to remove the hemicellulose and for structural breakdown of lignocellulosic biomass [14,16,18-21]. During these pretreatments, biomass is treated at different combinations of temperatures (100-290°C) and residence times (few seconds to several hours). During hydrothermal pretreatment, most of the hemicellulose is hydrolyzed to sugar monomers and becomes soluble. Some fraction of cellulose may be depolymerized into glucose. A fraction of lignin is dissolved and/or redistributed. Externally added acid (0.05-5%) and alkali act as catalysts during dilute acid and dilute alkali pretreatment process respectively. Hot water pretreatment is "auto catalyzed" process, where acetic acid released from hemicellulose and self ionization of water at elevated temperatures causes pH drop. High acid concentrations and/or high temperatures during dilute acid process can cause degradation of sugar monomers to furans, which are inhibitory to yeast during fermentation. In the dilute alkali process, alkali affects the lignin-carbohydrate linkages and removes acetyl groups of hemicellulose. This result in higher lignin removal during dilute alkali pretreatment compared to other pretreatments [9,10,22,23]. During steam explosion process, biomass is heated under pressure and the pressure is rapidly released in a flash tank. Rapid expansion of the steam causes an explosive breakdown of the biomass structure. A wide range of temperatures (160-290°C) and residence times have been investigated for this process [3,10]. The process has been found efficient using a combination of high temperature and short residence time or lower temperature and longer time [3,23]. A major advantage of steam explosion process is that it has been found effective on feedstock with large particle sizes, which can reduce the energy required for size reduction [3,24]. To quantify the effectiveness of pretreatment process, "combined severity (CS) factor" has been used in many studies. The CS factor relates variable process conditions such as temperature, time and pH (acid/alkali concentration) during pretreatment process [25-28]. Typical process conditions, mode of action and limitations of these pretreatment processes have been summarized in Table 1.

**Table 1:** Typical pretreatment process conditions of pretreatment*

	Dilute Acid	Dilute Alkali	Hot Water	Steam Explosion
Temperature (°C)	160-220	> 100	160-230	160-290

Pressure (MPa)	Saturated vapor pressure	Saturated vapor pressure	Up to 5	0.69-4.83

Solution (acid/alkali) concentration (%)	0.05-5	0.5-3	-	-

Residence Time	1-60 min	-Few minutes to several hours	12-60 min	Few seconds-several minutes

Mode of Action	-Hydrolysis of hemicellulose to soluble sugars -Alteration the lignin structure	-Solublization and extraction of lignin -Swelling of cellulose which increases internal surface area and separation of linkages between lignin and carbohydrates	-Water acts as dilute acid at high temperatures -Release of acetic acid and other acids from hemicellulose hydrolysis helps in further hydrolysis -Hydrolysis of hemicellulose to oligomers	-Water acts as dilute acid at high temperatures -Rapid release of pressure opens up the structure of biomass -Removal of hemicellulose

Challenges	-Process require Detoxification before fermentation -Acid is corrosive and hazardous so reactor material should be corrosion resistant	-Cost of alkali is very high as compared to other reagents -Longer residence times are required at low temperatures	-Process require high pressure to keep water in liquid state	-Formation of inhibitory compounds due to hemicellulose degradation

*Ref: [2,3,9,10,16,22,23,60,61]

Most of the models in recent years have been developed for lignocellulosic ethanol plants using dilute acid, steam explosion and AFEX pretreatment processes using laboratory scale data [19,29-31]. It is known that there are many tradeoffs in the design of commercial scale ethanol plants and these tradeoffs need to be understood to have an economically viable process [29,32,33]. A detailed process model which includes all unit operations from biomass handling to ethanol distillation can be helpful to perform the economic analysis of the whole process on commercial scale. Such models can aid in understanding the tradeoffs in capital costs, energy and water use in the process. The results (utilities requirement, energy and emissions) obtained from these models can also be used to analyze the environmental impact of the process using tools such as life cycle assessment.

Several researchers have used computer simulation process models for ethanol production from corn grain [34-36]. Wooley et al. [37] developed a process model using Aspen Plus for production of ethanol from lignocellulosic biomass using dilute acid pretreatment and enzymatic hydrolysis, which provided a base case for many further studies and cost estimate of ethanol production. A report from National Renewable Energy Laboratory (NREL) [19] provided the updated process model that included all details of operations such as feed handling, product recovery, wastewater treatment, in addition to all major unit operations. Using these models as base case, some other reports have also been published on process modeling of ethanol production using current and mature processing technologies [29,30]. While several researchers have developed process models for cellulosic ethanol for various individual pretreatment processes, it is difficult to compare the use of pretreatment technologies due to lack of a consistent process modeling framework for the underlying ethanol production process.

The aim of this work was to develop process models for several common pretreatment processes with a consistent underlying framework to investigate economic feasibility, compare energy consumption and sensitivity of the ethanol price to process parameters. Process models were developed considering four different pretreatment methods: dilute acid, dilute alkali, hot water and steam explosion for an ethanol plant with 250,000 metric ton/year of biomass processing capacity. All models were developed based on assumptions of existing and near - term cellulosic ethanol production technologies. Tall fescue grass was selected to compare different pretreatment methods for producing cellulosic ethanol, as significant amounts of this straw residue are produced (791,000 metric tons/year) in Pacific Northwest U.S. and its composition is similar to that of proposed dedicated herbaceous energy crops [18,38,39] . Sensitivity analysis was performed by varying the price of raw materials, fermentation efficiencies and efficiency of the electricity conversion to investigate their effect on ethanol price. In addition, tradeoffs in the energy and capital cost due to choice of downstream processing technologies were investigated.

## Methods

### Biomass

**Figure 2:**
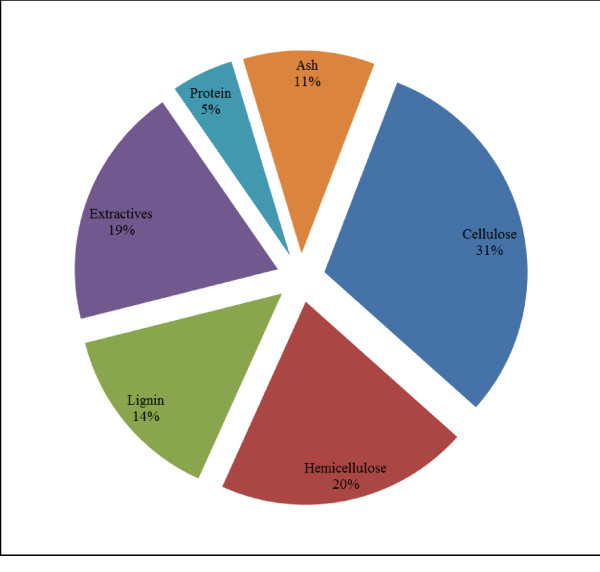
**Chemical composition of tall fescue grass straw**.

Grass straw is coproduct of grass seed production and a potential feedstock for biofuel production due to high cellulose content (up to 31%). Grass seed production is concentrated in the states of Oregon, Washington, and Idaho. In Oregon, about 0.88 million metric tons/year of grass straw is available as a co-product from grass seed production [38]. Tall fescue (*Festuca arundinacea Schreb*) straw is considered as a potential biomass for ethanol production as it is a major grass seed crop in Willamette valley, Oregon. Tall fescue (TF) seed production yields about 11.9 Mg/hectare of straw [38]. Straw from tall fescue contains about 31% cellulose, 20.2% hemicellulose and 14.4% lignin (Figure 2). Xylan is the main component of hemicellulose (82%) [18]. Heating value of biomass was calculated 13.2 MJ/dry kg biomass (lower heating value).

Perennial ryegrass (*Lolium perenne *L.) another grass seed crop can also be used in the same ethanol production plant. In laboratory experiments, composition and hydrolysis efficiencies for perennial ryegrass (PR) were similar to TF [18]. Area required to collect 250,000 Mg of grass straw was calculated using equation 1 [19].

(1) $$Area_{collect}=\frac{D_{straw}}{Y_{straw}*F_{cropland}*F_{avail.}*F_{collect}}$$

Where, D_straw _= Annual demand of straw, Mg; Y_straw _= Annual yield of straw, Mg/acre; F_cropland _= Fraction of area under fields; F_avail _= Fraction of farm land from which grass straw could be sold; F_collect _= Percentage of straw that can be collected from fields without affecting the soil quality

It was assumed that plant would be located in the center of farming areas and 40% of the area comes under infrastructure (roads, buildings etc.). It is important to note that all of the straw is not available for ethanol production as depending on the site specific agro-climatic and edaphic factors, some amount of straw is needed as mulch to maintain soil organic carbon content and soil health. The values of F_avail _and F_collect _were assumed to be 0.75 and 0.5 respectively. Considering 11.8 Mg/ha (4.8 Mg/acre) annual grass straw yield (Y_straw_) [38], it was calculated that 230,966 acres would be required to collect 250,000 metric ton of grass straw annually. Assuming the plant to be located in the center of grass seed farmland, a distance (radius) of 17.25 km around the plant is sufficient to supply the required amount of straw.

### Model development

The process models were developed using Super Pro Designer (Intelligen, Inc., Scotch Plains, NJ) for an ethanol plant with processing capacity of 250,000 metric ton biomass/year. Ethanol production process was divided into four sections: front end operations (e.g. cleaning and size reduction of biomass), pretreatment of biomass, simultaneous saccharification and co-fermentation (SSCoF) and downstream processing. Downstream processing consisted of anhydrous ethanol recovery using distillation and molecular sieves, co-product (lignin) recovery/utilization to generate steam and electricity, and waste water treatment. Only the pretreatment section was different among different models and other operations were same in all four models. As these models were developed for the pretreatments using same underlying model and assumptions, their performance could be compared on a consistent basis. Most common models [34-37] in literature are the so called factored estimate models and are accurate up to 30% [40]. While details of minor equipment such as valves and pumps were not included in these models, factors based on total equipment cost are used to model the process economics. Such models are useful to establish differences in technologies but cannot be used for making decisions regarding financing/construction of plants. The process parameters and efficiencies used in the current models and other literature studies are summarized in Table 2.

**Table 2:** Summary of process conditions and efficiencies used in current models and other studies

	Parameters	Dilute Acid					Hot Water			Dilute Alkali	Steam Explosion
		**Present Study**	**Laser et al**. [30]	**Aden et al**. [19]	**Kazi et al**. [29]	**Eggeman and Elander **[31]	**Present Study**	**Kazi et al**. [29]	**Eggeman and Elander **[31]	**Present Study**	**Present Study**

**Biomass**	**Biomass**	**Tall Fescue**	**Switchgrass**	**Corn Stover**	**Corn Stover**	**Corn Stover**	**Tall Fescue**	**Corn Stover**	**Corn Stover**	**Tall Fescue**	**Tall Fescue**

Biomass Processing	Feedstock Rate (dry Mg/day)	704.5	4535	2000	2000	2000	704.5	2000	2000	704.5	704.5

Pretreatment	Temperature (°C)	180	190	190	190	140	180	190	180	180	180
	
	Pressure (bar)	11	12.3	12.3	11.8	-	11	12.5		11	11
	
	Residence Time (min)	15	2	2	2	-	15	5	5	15	15
	
	Solid Loading (%)	20	30	30	29.6	-	20	12.9	13.9	20	30
	
	Acid/Alkali Concentration (%)	1	1.1	1.1	1.9	1	0	0		1	-
	
	Cellulose + 0.111 H_2_O→ 1.111 Glucose (%)	13.04	6.5	7	6.3	8^c^	0.43	0.32	4.5^c^	0.29	5
	
	Xylans + 0.136 H_2_O → 1.136 Xylose (%)	60.26	85	90	82.5	90.2^c^	70	2.39	50.8^c^	0.72	70
	
	Lignin → Soluble Lignin (%)	5	5	5	10	-	5	5		25	5
	
	Xylose → 0.64 Furfural + 0.36 H_2_O (%)	5	5^a^	5^a^	10^a^	-	2.5	0^a^	-	0.01	15
	
	Glucose → 0.7 HMF + 0.3 H_2_O (%)	5	-	-	3^b^	-	2.5	0^b^		0.01	15

Hydrolysis and Fermentation	Bioconversion Method	SSCoF^d^	SSF^e^	SSCoF	SSCoF	-	SSCoF	SSCoF		SSCoF	SSCoF
	
	Temperature (°C)	35	37	65 (Sacchaarification) 41 (Co-fermentation)	32	-	35	32		35	35
	
	Enzyme Loading (FPU/g cellulose)	15	15	12	18^f^	15	15	18^f^	15	15	15
	
	Time (Saccharification + Fermentation) (days)	5	7	3 (1.5 + 1.5)	7 (5+2)	-	5	7 (5+2)		5	5
	
	Cellulose + 0.111 H2O→ 1.111 Glucose	79	80	90	91.09	83.8^c^	78.5	89.97	90^c^	84.75	70
	
	Xylans + 0.136 H_2_O → 1.136 Xylose (%)	80	80	-	57.13	55.1^c^	80	56.6	64.63^c^	80	80
	
	Glucose → 0.489 CO_2 _+ 0.511 Ethanol (%)	95	90	95	95		95	95		95	95
	
	Xylose → 0.489 CO_2 _+ 0.511 Ethanol (%)	70	90	85	75.6		70	75.6		70	70
	
	Cellulase → 0.1 Protein Soluble + 0.9 water (%)	99	-	-	-	-	99	-	-	99	99

Ethanol Recovery	Ethanol Recovery (%)	98.76	98.8	98.9	-	-	98.76	-	-	98.76	98.76

^a ^Percentage conversion of xylans to furfural

^b ^Percentage conversion of cellulose to furfural

^c ^The efficiencies are based on cumulative sugars (oligomers+monomers)

^d ^SSCoF - Simultaneous saccharification and Co-fermentation

^e ^SSF - Simultaneous saccharification and fermentation

^f ^31.3 mg protein/g cellulose (600 FPU/g protein)

#### Front end operations

Grass straw is transported to plant over a distance of about 17 km in truck trailers in the form of bales. Grass straw bales are assumed to be predominantly stored in barns. Biomass is transported from the storage locations to the plant on belt conveyors. In the cleaning step, biomass is washed with water and impurities such as soil and metal debris are separated. The water containing impurities is diverted to waste water treatment plant and 70% of water is recycled back. Particle size reduction is accomplished in a knife mill.

#### Pretreatment of biomass

Models were developed for four different pretreatments: dilute acid, dilute alkali, hot water and steam explosion. These pretreatments methods have been investigated for different feedstocks by many researchers. The process conditions, principles, advantages and limitations of these pretreatment processes have been discussed in many review papers [2,3,9,10,41,42]. Dilute acid, dilute alkali and hot water pretreatment processes were modeled for 20% solid loading, whereas 30% solid loading was used for steam explosion.

**Figure 3:**
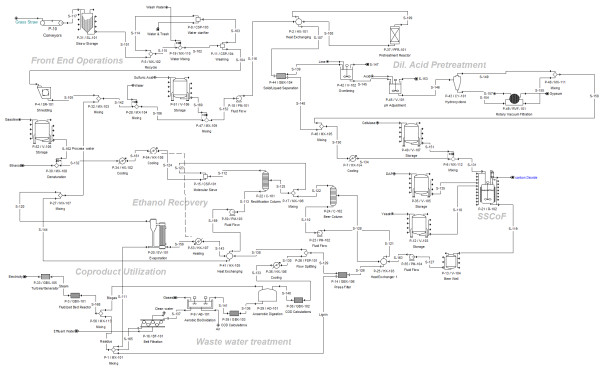
**Process model of ethanol production from grass straw using dilute acid pretreatment**. Figure illustrates the all unit operations and equipments used in the model development.

The ethanol production process using dilute acid pretreatment and SSCoF is shown in Figure 3. During this process, grass straw is treated in a dilute H_2_SO_4 _solution (1% w/w of solution) at 180°C with a 15 min residence time in the reactor. Amount of sugars removed during the process was calculated based on previous lab scale studies [18]. The heated slurry is immediately cooled by exchanging heat with input stream to reactor. The pretreatment process is indicated by a single reactor as there is not much information available regarding commercial scale pretreatment reactor design. In practice this process would contain a series of equipment: screw conveyors, tanks and reactor. Solid and liquid portions are separated using pneumapress pressure filter to facilitate the detoxification [19]. Overliming process is used as the detoxification or 'conditioning' step. During the overliming process, liquid fraction is adjusted to 10.0 pH using Ca(OH)_2_. Subsequently, the pH is adjusted to 5.0-6.0 pH by adding H_2_SO_4 _[19]. The liquid stream is combined with the solid fraction and is fed to SSCoF process.

During the dilute alkali process, dilute NaOH (1% w/w of solution) is used to treat the biomass at 180°C with a 15 min residence time. It was observed from our previous laboratory studies, that only a small amount of hemicellulose is broken down during this process [18]. It was also observed that solubilization of lignin makes it difficult to separate liquid and solid streams. So, during developing the model of this process, it was assumed that whole slurry would be fed to SSCoF process after adjusting the pH with dilute sulfuric acid.

During hot water treatment, also known as 'hydrothermal treatment', biomass is treated with liquid water at temperature of 180°C and 15 min residence time (Table 2). Based on literature values, 70% hemicellulose hydrolysis was assumed during hot water pretreatment for model simulations.

**Figure 4:**
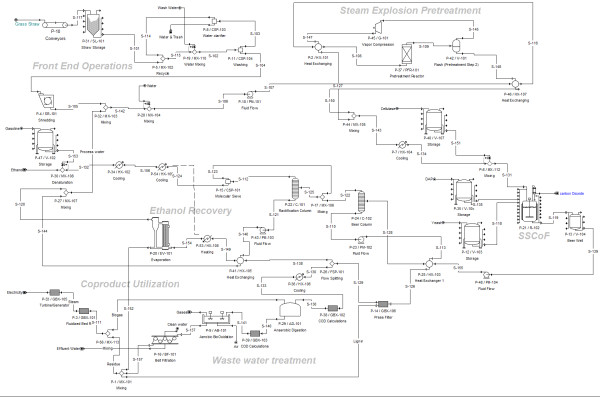
**Process model of ethanol production from grass straw using steam explosion pretreatment**. Figure illustrates the all unit operations and equipments used in the model development.

During steam explosion process, biomass is heated to high temperature under pressure and the pressure is instantly released in a flash tank. A temperature of 180°C and 5 min residence time was assumed in for pretreatment process. Vapors from the flash tank are compressed using vapor compressor and this heat is used to increase the temperature of stream coming to pretreatment reactor. The process model using steam explosion pretreatment is presented Figure 4.

#### Hydrolysis and fermentation

The pretreated grass straw was hydrolyzed using commercial enzymes (blend of cellulases and hemicellulases) at an enzyme loading of 15 FPU/g of cellulose [21]. The activity of enzymes was assumed to be 600 FPU/g of protein and 10% protein in broth purchased from market (60 FPU/ml enzymes mixture) [29]. SSCoF process includes enzymatic hydrolysis of cellulose and hemicellulose; simultaneous fermentation of resulting hexose and pentose sugars. Presently, glucose fermentation is efficient and well-developed commercial scale technology; however co-fermentation of glucose along with other sugars has been tested successfully on laboratory and pilot scale only [19,43]. The organism tested for co-fermentation of glucose and xylose is genetically modified *Zymomonas mobilis *[2,19,44,45]. Cellulose hydrolysis efficiencies for dilute acid, dilute alkali and hot water pretreated grass straw were obtained from laboratory studies [18]. The cellulose hydrolysis efficiency of 70% was assumed for straw pretreated with steam explosion [23,46]. Hemicellulose hydrolysis efficiency (in enzymatic hydrolysis) was assumed as 80% in all cases. The fermentation efficiencies of glucose and xylose were assumed to be 95% and 70% respectively.

#### Downstream processing

Fermented slurry is stored in beer well for four hours, which allows decoupling of the batch SSCoF process and the continuous distillation process. Ethanol is subsequently recovered using a combination of distillation columns and molecular sieves. The distillation design was based on NREL 2002 report [19,30]. The first distillation column in the process, called a beer column, separates ethanol as overhead vapors. Bottom effluent from this column contains mostly lignin, insoluble proteins and other non-fermentables. Ethanol enriched vapors from the beer column are further enriched in the rectification and stripper columns. Resulting azeotropic mixture of water and ethanol from the rectification column is separated using molecular sieves to produce anhydrous ethanol which is denatured by addition of gasoline. The bottom effluent of beer column is passed through a pneumapress filter that separates it into two streams: a solid stream which contains most of the lignin and a liquid stream containing most of the water and soluble solids. The lignin rich solid stream is combusted in fluidized bed combustor for steam generation [19,29,30]. The solid stream contains about 50% moisture in case of dilute acid, dilute alkali and hot water, and about 38% moisture for steam explosion. The liquid stream is divided into two parts, one fraction (25%) liquid stream is treated in waste water treatment plant and remaining liquid is concentrated in multiple-effect evaporator. The condensate stream from evaporator containing water (> 99%) is recycled back as process water. The concentrated syrup after evaporation, containing about 40% (wet basis) solids is fed to combustor together with the sludge from waste water treatment. This stream (mixture of lignin stream, evaporator concentrates and sludge) with about 55% moisture is burned in fluidized bed combustors to produce process steam. In general steam produced from lignin fraction is more than the steam requirement of plant, so the excess steam can be used to generate electricity. The excess electricity can be sold to grid [19,29,30]. The waste water treatment system consists of anaerobic digestion followed by aerobic digestion [19,47]. The liquid stream is treated in anaerobic digester using mesophilic bacteria for 10 days, which convert volatile solids to mixture of methane and carbon dioxide (biogas). Biogas produced from anaerobic digester is burned in the combustor along with lignin residue stream to produce steam. The amount of biogas generated was calculated on basis of chemical oxygen demand (COD). Methane is produced from water treatment at the rate of 0.35 m^3 ^per kg of COD removed (0.239 kg/kg COD) [47]. One mole of CO_2 _is produced for every three moles of methane (0.22 kg/kg COD) [19]. Cell mass is produced at the rate of 0.03 kg/kg of COD removed [19,47]. For COD calculations, 90% degradation was assumed for soluble sugars, organic acids, ethanol, enzymes and 50% degradation for residual carbohydrates, extractives and water soluble lignin [47]. Insoluble lignin was not considered in COD calculations. Treated water containing sludge, residues and water is subsequently fed to aerobic digester, with a residence time of 6 days. The sludge from aerobic digester is fed to combustor as described earlier.

### Economic analysis

Costs of most of the equipment (pretreatment reactor, pneumapress filter, shredder, fermenters, fluidized bed reactor, turbine/generator) were calculated based on cost models based of 2002 NREL dilute acid model [19], Laser et al. [30] and Corn ethanol plant models [34,35]. Built-in cost models in SuperPro designer was used to determine cost of some equipment. Costs of heat exchangers and some other equipment were estimated from equipment cost database [48]. The cost of new equipment for different sizes was calculated using the exponential scaling equation (Eqn. 2) [19].

(2) $$New\;Cost=Original\;Cost{\left(\frac{New\;Size}{Original\;size}\right)}^{exp.}$$

Costs of utilities and other consumables were either estimated based on recent studies [29,30,35], built in models or according to current purchase costs from suppliers. Other than purchase costs, SuperPro Designer estimates the additional cost of installation, piping, electrical, insulation, design work, and buildings for facility. These all costs are accounted as the direct cost (DC). Installation costs were calculated by considering in installation cost factors and purchase cost of each equipment. The installation factors were obtained from other techno-economic studies [19,29,30]. Other than direct cost, there are some indirect costs, which include engineering costs (estimated to be 5% of DC) and construction costs (10% of DC). Other than these costs, contractors' fees and contingency costs were estimated as 5% and 10% of sum of direct cost and indirect cost. Sum of all these costs is direct fixed cost (DFC). Dollar values used in the analysis were for cost year 2010. Construction period was assumed as 24 months, with start-up time of 6 months. Project life and a depreciation period of 20 and 10 years respectively were considered with a 5% salvage value. Direct fixed cost (expenditure) was distributed over first three years (30%, 40% and 30% in first, second and third year respectively).

## Results and discussion

Ethanol production processes using grass straw as feedstock and four different pretreatment technologies were simulated in SuperPro Designer with an annual biomass processing capacity of 250,000 metric tons/year. The ethanol production capacities were calculated as 59.66, 59.47, 59.35 and 53.53 million L (15.76, 15.71, 15.68 and 14. 14 million gal) for plants using dilute acid, dilute alkali, hot water and steam explosion pretreatments respectively (Table 3). Estimated ethanol yields were 256.62, 255.8, 255.3 and 230.2 L/dry metric ton of biomass for plants using dilute acid, dilute alkali, hot water and steam explosion pretreatments respectively. Ethanol yields were relatively low for steam explosion case due to comparatively low (70%) cellulose hydrolysis efficiency.

**Table 3:** Overall economics of the ethanol plant with 25 MT of biomass processing (2010 prices)

	Dilute Acid	Dilute Alkali	Hot Water	Steam Explosion
Total Investment (MM$)	114.63	102.77	101.89	91.36

Operating Cost (MM$/yr)	50.06	52.70	48.10	45.83

Ethanol Revenue (MM$/yr)	65.41	65.21	65.07	58.64

Ethanol (MM gal/yr)	15.76	15.71	15.68	14.14

Ethanol Unit Cost ($/gal)	3.18	3.35	3.07	3.24

### Overall economics

**Figure 5:**
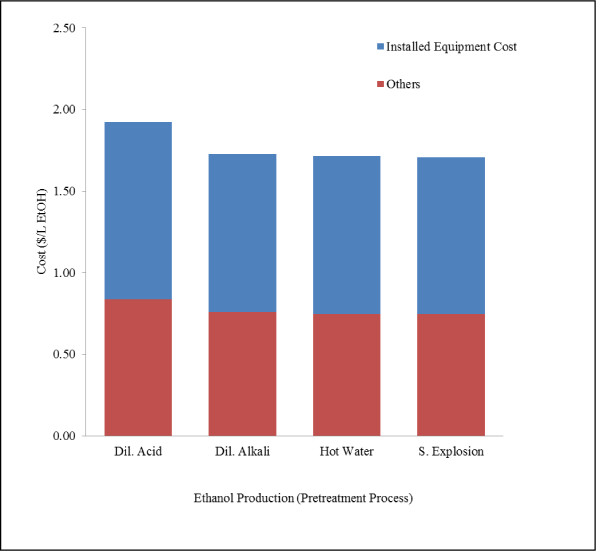
**Capital cost for ethanol production from grass straw using different pretreatment processes**. Figure shows the capital cost (direct fixed cost) per liter of ethanol. Figure also illustrates the breakdown of installed equipment cost and other costs. Other includes piping, electrical, insulation, design work, and buildings and construction, engineering costs, contractors' fees and contingency.

Capital costs were estimated to be $ 1.92, 1.73, 1.72 and 1.71 per L of ethanol produced for plants using dilute acid, dilute alkali, hot water and steam explosion pretreatment process respectively (Table 3). Capital cost includes piping, insulation, buildings, and other indirect costs other than installed equipment cost. The breakdown of installed equipments costs and other costs is shown in Figure 5. Among all the models, capital cost was found highest for dilute acid process ($114.63 MM). This was due to additional investment in equipment required for solid and liquid streams after pretreatment (pneumapress) and detoxification (overliming tank, hydrocyclone and rotary filter). Eggeman and Elander [31] conducted the economic analysis of ethanol production plant with capacity of 189.5 MM L/year using corn stover as substrate and different pretreatment technologies. They estimated about $0.98/L ethanol ($208.6 MM total) and $1.2/L ethanol ($200.9 MM total) capital investment to start the plants with dilute acid and hot water pretreatments respectively. Laser et al. [30] developed process model for ethanol production from switchgrass using a base case scenario of dilute acid pretreatment using NREL [19] design and estimated capital investment of $1.2/L ethanol (603.8 $MM for 133.3 MM gal ethanol/year). The capital costs obtained from dilute acid and hot water pretreatment process in current study were similar to those obtained by Kazi et al. [29] for corn stover ($1.86/L and $2.2/L ethanol for dilute acid and hot water pretreatment respectively). Kazi et al. [29] obtained low capital cost per liter of ethanol for dilute acid process as compared to hot water pretreatment because of relatively higher ethanol yield (289 L/Mg biomass for dilute acid vs. 211 L/Mg biomass for hot water pretreatment). The capital costs of boiler were found in the range of $149 - 182.4/kWh thermal energy produced for different pretreatment models, which were in agreement with values provided in literature ($80-340/kWh) [49]. Details of equipment cost and capital cost calculations are provided in the supplementary material (Additional file 1).

**Figure 6:**
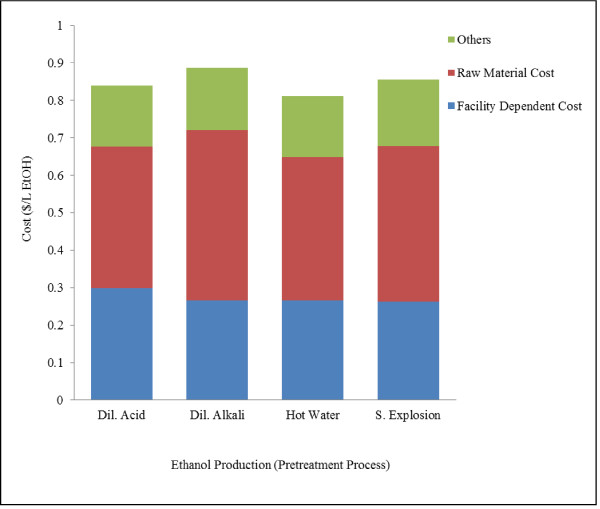
**Operating cost for ethanol production from grass straw using different pretreatment processes**. Figure shows the operating cost per liter of ethanol. Figure also illustrates the breakdown of facility dependent, raw material and other costs. Other costs include labor, utilities and waste disposal.

Ethanol production costs were estimated to be $0.84, 0.89, 0.81 and 0.86 per L ethanol for plants using dilute acid, dilute alkali, hot water and steam explosion pretreatment process respectively. Operating costs included facility dependent costs, raw material costs, utility costs and labor costs. Breakdown of facility dependent costs, raw material costs and other costs for all processes is illustrated in Figure 6. Ethanol production costs for dilute acid and hot water pretreatments were found similar to the values calculated by Kazi et al. [29] ($0.91/L ethanol ($1.36/L gasoline equivalent) for dilute acid and $1.88/L ethanol ($1.77/L gasoline equivalent) for hot water treatment). However, these values were higher than $0.28/L ($1.07/gal) (year 2002 dollars) reported by Aden et al. and $0.45/L ($1.71/gal) ethanol reported by Laser et al. [30] for plant using dilute acid pretreatment process. NREL study [19] was conducted and plant model was developed keeping target price of $1.07/gal ethanol. Ethanol production cost in the present study is higher than the NREL studies due to low hydrolysis and fermentation efficiencies of pentose sugars, high cost of biomass, high enzyme loadings and smaller plant size used in development of models in the present study. Facility dependent costs accounted for about 30-35% of operating costs, which were found higher in this study due to lower solid loading in dilute acid, dilute alkali and hot water pretreatment (20% as compared to 30% in other studies). Among all the models, ethanol price was found highest for dilute alkali process due to relatively high purchase cost of alkali ($0.45/kg for NaOH vs. $0.035/kg sulfuric acid). Cost of ethanol was found high in case of steam explosion pretreatment process due to low cellulose hydrolysis yield (70%).

It is important to note that the values used in the model development were chosen on basis of laboratory studies and literature surveys. Lowest capital cost for steam explosion case was because of assumption of high solid loading (30% w/w) during pretreatment and hydrolysis processes.

### Raw materials

The costs associated with bulk materials used in the whole processes are presented in Table 4. Grass straw (20.95-23.35 ¢/L ethanol) and cellulase enzymes (13.5-16.5 ¢/L ethanol) were the main contributors in the total material cost. Because of high cost of enzymes, enzymatic hydrolysis process has been reported as main cost affecting unit process in the ethanol production plant [6,50]. In the cost analysis model by Eggeman and Elander [31], cost of enzymes was assumed to be $0.04/L ($0.15/gal) of ethanol, as an estimate of a reasonable cost in future refineries. Commercial enzyme producing companies such as Novozymes [51] have estimated the enzyme cost of about $0.13/L ($0.5/gal) of ethanol production. The cost of enzyme broth (activity of 60 FPU/ml broth or 600 FPU/mg protein) used in this model was set to $0.517/kg of enzyme broth [29]. In case of process using dilute acid pretreatment, grass straw cost of about 20.95 ¢/L (79.3 ¢/gal) of ethanol was estimated, which was higher than those of corn stover (33.4 ¢/gal, Aden et al. [19]) and switchgrass (58.3 ¢/gal, Laser et al. [30]). This can be attributed to relatively low cellulose and hemicellulose contents and low pentose fermentation yields assumed in this model. Another reason for high cost could be that process efficiencies and assumptions used in the models were based on actual experimental data rather than theoretical assumptions.

**Table 4:** Overall bulk materials used in ethanol making process (2010 prices)

		Dilute Acid		Dilute Alkali		Hot Water		Steam Explosion	
**Material**	**Unit Cost ($/kg)**	**Annual Cost ($1000×)**	**Cost (¢/L EtOH)**	**Annual Cost ($)**	**Cost (¢/L EtOH)**	**Annual Cost ($1000×)**	**Cost (¢/L EtOH)**	**Annual Cost ($)**	**Cost (¢/L EtOH)**

Water	0.0003	106.69	0.18	108.21	0.18	104.04	0.18	70.05	0.13

Tall Fescue	0.0500	12500.00	20.95	12500.00	21.02	12500.00	21.06	12500.00	23.35

Sulfuric Acid	0.0350	429.12	0.72	99.79	0.17	0	0.00	0	0.00

Ca Hydroxide	0.1000	597.09	1.00	0	0.00	0	0.00	0	0.00

DAP	0.2100	16.632	0.03	16.63	0.03	16.63	0.03	16.63	0.03

Cellulase	0.5170	8066.60	13.52	9279.01	15.60	9265.98	15.61	8840.70	16.52

Yeast	2.3000	455.40	0.76	455.40	0.77	455.40	0.77	455.40	0.85

Sodium Hydroxide	0.4500	0.00	0.00	4203.80	7.07	0.00	0.00	0	0.00

Gasoline	0.8000	390.53	0.65	389.29	0.65	388.48	0.65	350.10	0.65

### Utilities

Bioethanol facilities require large amounts of process steam at various process pressures: low pressure (LP) steam (152°C and 502 kPa) and high pressure (HP) steam (242°C and 3464 kPa). HP steam is usually recycled for LP applications before the condensate is returned to boilers in order to extract the maximum heating capacity from the steam, which was assumed 50% while calculating steam requirement. Amount and costs of utilities (steam, cooling water, electricity etc.) calculated from all four models are summarized in Table 5 and 6. Amount of steam generated from lignin stream was more than the plant requirement in all cases and hence the cost of steam was adjusted to zero. The revenue from lignin sales was also set to zero as all of the lignin stream will be used for on-site steam and electricity generation. The values of electricity and steam used in ethanol plant using dilute acid pretreatment process were found to be 0.56 KWh/L and 6.3 kg/L of ethanol produced, which are comparable to 0.38 KWh/L (1.42 KWh/gal) and 4.42 kg/L (16.7 kg/gal) of ethanol calculated by Aden et al. [19]. It should be noted that electricity values presented in Tables 5 and 6 did not account for electricity required for cooling and chilled water production, however the energy required was accounted in the process energy calculations.

**Table 5:** Overall utilities used in ethanol making process using dilute acid and dilute alkali pretreatment (2010 prices)

	Dilute Acid			Dilute Alkali		
Utility	Amount (kg/L EtOH)	Annual cost $ (%)	Cost (¢/L EtOH)	Amount (kg/L EtOH)	Annual cost $ (%)	Cost (¢/L EtOH)
Electricity (KWh)^a^	0.56	2334792 (55.4)	3.91	0.52	2163214 (53.5)	3.64
Steam	5.91	0	0.00	5.82	0	0.00
Cooling Water	500.24	1492305 (35.4)	2.50	500.50	1488313 (36.8)	2.50
Chilled Water	0.73	17367 (0.4)	0.03	0.84	19977 (0.5)	0.03
CT Water	88.76	370702 (8.8)	0.62	89.04	370702 (9.2)	0.62
Steam (High P)	0.42	0	0.00	0.42	0	0.00

^a ^These electricity values did not account for electricity required for cooling and chilled water

**Table 6:** Overall utilities used in ethanol making process using hot water and steam explosion pretreatment (2010 prices)

	Hot water			Steam Explosion		
**Utility**	**Amount (kg/L EtOH)**	**Annual cost $ (%)**	**Cost (¢/L EtOH)**	**Amount (kg/L EtOH)**	**Annual cost $ (%)**	**Cost (¢/L EtOH)**

Electricity (KWh)^a^	0.52	2161442 (50.9)	3.64	0.58	2165478 (59.7)	4.05

Steam	6.01	0.00	0.00	4.03	0.00	0.00

Cooling Water	570.00	1691500 (39.9)	2.85	401.44	1074393 (29.6)	2.00

Chilled Water	0.84	19949 (0.5)	0.03	0.89	19034 (0.5)	0.04

CT Water	89.23	370702 (8.7)	0.63	98.94	370702 (10.2)	0.69

Steam (High P)	0.42	0.00	0.00	0.62	0.00	0.00

^a ^These electricity values did not account for electricity required for cooling and chilled water

The steam energy used for production of ethanol ranged from 13.55 to 19.33 MJ/L of ethanol (Table 7). The residue stream (lignin stream, evaporator concentrate and sludge) along with biogas from anaerobic digestion is used to produce steam by burning in fluidized bed combustors. Amount of steam that could be generated was calculated based on the heating values of different stream constituents (soluble sugars, cellulose, hemicellulose, lignin and protein) and biogas heating value. While calculating the energy from lignin stream, energy required to remove the moisture (~55%) was deducted from total available energy. Biomass boiler efficiency varies 50-85% depending on the type and moisture content of the feedstock [49,52], therefore a 75% boiler efficiency was used for all models. Lignin energy ranged from 27.96 to 34.82 MJ/L of ethanol produced depending on the pretreatment process used. Excess lignin energy (steam) was assumed to be used for electricity production with 30% conversion efficiency from biomass energy. Potential of electricity production ranged from 0.77 kWh/L for dilute acid/hot water pretreatment to 1.78 kWh/L of ethanol for steam explosion process. Significant amount of electricity energy was also used in the form of cooling and chilled water to dissipate heat during various operations. Cooling water requirement was found minimum for steam explosion (401.4 kg/L of ethanol) due to higher solid loading which leads to reduced flow rates of streams and hence lower energy for cooling. Requirement of utilities to produce one liter of ethanol using different processes are presented in Table 7. Fresh water requirement during production of fuels is a growing concern all over the world. Water requirements were estimated to be 5.96, 6.07, 5.84 and 4.36 kg/L of ethanol produced using dilute acid, dilute alkali, hot water and steam explosion pretreatment.

**Table 7:** Steam demand and lignin energy available for electricity production

Pretreatment Process	Steam demand (kJ/L EtOH)	**Steam demand (kJ/kJ biomass)**^**a**^	Lignin Energy (kJ/L EtOH)	**Lignin Energy (kJ/kJ biomass)**^**a**^	Excess Lignin Energy (kJ/L EtOH)	**Electricity Produced (kWh/L EtOH)**^**b**^
Dilute Acid	19010	0.369	28232	0.548	9223	0.77
Dilute Alkali	18737	0.363	27967	0.542	9229	0.77
Hot Water	19333	0.374	29138	0.563	9804	0.82
Steam Explosion	13508	0.235	34819	0.607	21310	1.78

^a^Biomass heating value: 13.21 MJ/kg (Lower heating value)

^b^Assuming 30% efficiency of lignin energy to electricity conversion

### Comparison to previous studies

**Figure 7:**
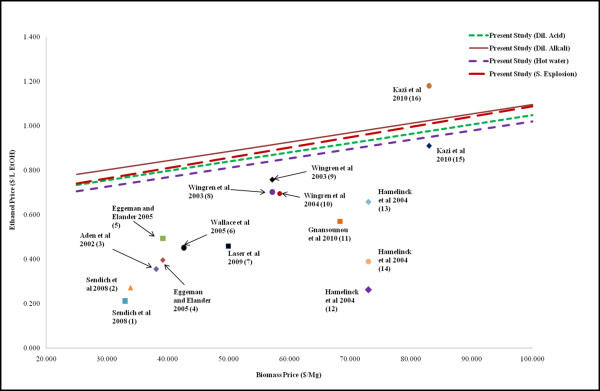
**Ethanol cost estimations from current models and previous techno-economic studies on ethanol production process (2010 prices)**. (1) Sendich et al. [53] - Consolidated bio-processing (CBP), (2) Sendich et al. [53] - Simultaneous saccharification and co-fermentation (SSCoF), (3) Aden et al. [19], (4) Eggeman and Elander [31] - Dilute acid pretreatment, (5) Eggeman and Elander [31] - Hot water pretreatment, (6) Wallace et al. [54], (7) Laser et al. [30] - Base case- dilute acid pretreatment, (8) Wingren et al. [55] - Separate hydrolysis and fermentation, (9) Wingren et al. [55] - Simultaneous saccharification and fermentation (SSF), (10) Wingren et al. [56], (11) Gnansounou et al. [32](12) Hamelinck et al. [57] - Long term technology (Hot water pretreatment, CBP), (13) Hamelinck et al. [57] - Short term technology (Dilute acid pretreatment, SSF), (14) Hamelinck et al. [57] - Middle term technology (Steam explosion pretreatment, SSCoF), (15) Kazi et al. [29] - Dilute acid pretreatment, (16) Kazi et al. [29] - Hot water pretreatment.

There is a large body of literature with techno-economic analysis for lignocellulosic ethanol process from various feedstocks [19,29-32,53-57]. Many of the models were derived from the two NREL studies by Wooley et al. [37] and Aden et al. [19]. A comparison of the ethanol prices presented by various authors to results from present study indicates that the generally ethanol price correlates well with feedstock price (Figure 7). Similar trends were observed by Kazi et al. [29]. Ethanol prices from this study are higher than most studies except for a few cases. In addition to feedstock price, higher ethanol prices predicted in the present study can be attributed to the differences in feedstocks, pretreatment technology, onsite/off site cellulase enzyme production, SSF/SSCoF/Consolidated bioprocessing, short/medium/mature technology scenarios and solid content of process streams. For example, ethanol price from the present study is higher than Kazi et al. [29] for dilute acid pretreatment ($0.977/L vs. $0.91/L) due to lower solid content (20% in present study vs. 30% in Kazi et al.) used in the pretreatment process. However, lower ethanol prices in the present study ($0.975/L in present study vs. $1.18/L) for hot water pretreatment process could be attributed to a greater effect of 8.1% lower solid content (20% in present study vs. 12.9%) compared to 11.47% higher enzymatic hydrolysis yields (78.5% in present study vs. 89.97% in Kazi et al [29]) on ethanol prices. This difference in enzymatic yields can be attributed to difference in the feedstocks: corn stover (Kazi et al. [29]) and Tall Fescue (present study).

### Sensitivity analysis

A wide range of values for cost of ethanol (Figure 7), capital costs, utilities and productivities are reported in literature [19,29-32,57]. In this context, it is of critical importance to understand the sensitivity of the results to various assumptions in the models. Sensitivity analysis was performed for some of the important parameters assumed in the models such as biomass price and pentose fermentation efficiency.

Biomass price was a major contributor in total material cost. The impact of biomass price on production cost of ethanol was investigated for all models (Figure 7). Biomass price of $50/metric ton was assumed for development of actual models, which resulted in biomass cost of $0.79 and $0.89 per gallon of ethanol for dilute acid and steam explosion pretreatment respectively. The sensitivity of biomass price on ethanol cost was studied by changing the grass straw price from $25 to $100/metric ton. By reducing the grass straw price from $50 to $25/metric ton, the ethanol production cost decreased by 12.6 and 13.6% for processes using dilute acid and steam explosion respectively.

**Figure 8:**
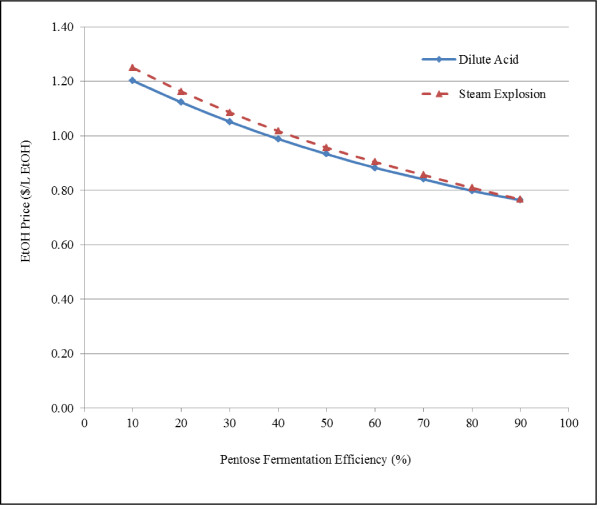
**Effect of pentose fermentation efficiency on cost of ethanol for dilute acid and steam explosion pretreatment process**.

One of the major challenges in the cellulosic ethanol production is fermentation of pentose sugars, which are significant part of biomass. Efficiency of xylose utilization is low for many microorganisms [58,59]. Pentose fermentation efficiency of 70% was assumed for model simulations in all cases. The sensitivity of pentose fermentation on ethanol price was investigated by varying the efficiency from 10 to 90% for dilute acid and steam explosion pretreatment processes (Figure 8). At 10% fermentation efficiency, the cost of ethanol production for dilute acid and steam explosion was $1.202/L and $1.250/L of ethanol respectively, which decreased to $0.764/L and $0.766/L of ethanol respectively as the pentose fermentation efficiency increases to 90%.

**Figure 9:**
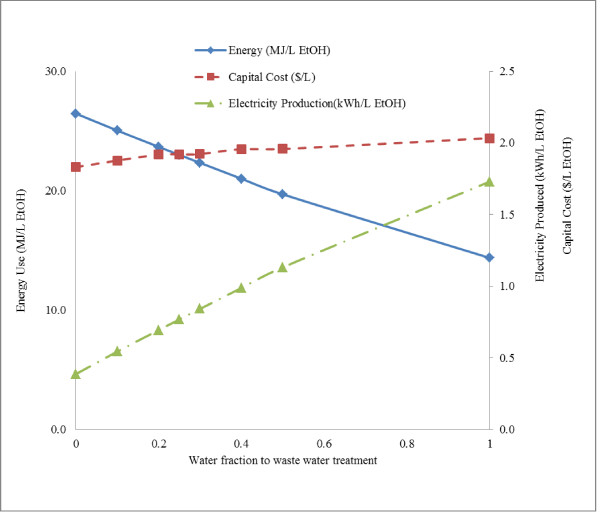
**Impact of process water treatment on energy use, capital cost and electricity production**. Figure illustrates the tradeoff between energy use and capital cost as the liquid stream after filter press is distributed among waste water treatment and evaporator. Blue and red lines show the total process energy and capital cost in the process per liter of ethanol. Green line shows the electricity produced from the excess steam (after using process steam) produced from lignin energy.

Interdependence of capital cost and energy use in the ethanol production process was investigated for dilute acid pretreatment by varying the percentage of water diverted to anaerobic digestion process and multiple effect evaporators. While multiple effect evaporators incur lower capital costs, they also lead to increased energy use compared to anaerobic digesters. Hence the percentage of process water diverted to multiple effect evaporators and anaerobic digester represents a tradeoff in energy and capital costs. For dilute acid process, energy use showed a 14.38% decrease (23.0 to 19.70 MJ/L ethanol) as the liquid stream from filter press to anaerobic digester increased from 25 to 50% (Figure 9). Correspondingly the unit price of ethanol and unit capital cost increased from $0.84 to $0.85/L ethanol and $1.92 to $1.96/L ethanol. Since the electricity is produced from excess steam that is not used in the process, electricity production decreases with increasing energy use due to reduction in available steam for electricity production. Electricity production decreases from 1.13 to 0.77 kWh/L ethanol as the liquid stream from filter press to anaerobic digester decreased from 50 to 25%; correspondingly, process energy use increases from 19.70 to 23.0 MJ/L ethanol. The results demonstrate the importance of addressing the tradeoffs in capital costs, pretreatment and downstream processing technologies in addressing the energy and capital costs in cellulosic ethanol plants. The electricity production efficiency from biomass energy is an important assumption in the process. The efficiency was assumed to be 30% in the present study. Variation in electricity production efficiency is possible due to improvements in technology or maintenance related issues. The electricity production was found 0.64 kWh/L and 1.03 kWh/L ethanol at 25% and 40% conversion efficiencies respectively. At 40% conversion efficiency, electric energy produced from extra steam (1.03 kWh/L ethanol) will be sufficient to provide electricity required for plant (0.92 kWh/L ethanol), while it would not be adequate at 25% conversion efficiency.

## Conclusions

Four process models for ethanol production plant with 250,000 metric ton/year grass straw processing capacity using different pretreatment technologies were developed using Super Pro Designer. The capital cost of the ethanol production plant ranged from 91.36 MM$ for steam explosion pretreatment to 114.63 MM$ for dilute acid pretreatment processes. The capital cost was found minimum for steam explosion because of high solid loading assumption during pretreatment and hydrolysis processes. The ethanol production costs for plants using dilute acid, dilute alkali, hot water and steam explosion pretreatment process were estimated as $0.84, 0.89, 0.81 and 0.86 per liter of ethanol respectively. Unit ethanol production cost was lowest for hot water pretreatment as no chemicals were required for pretreatment and the hydrolysis yields were similar to other pretreatment methods. Biomass (46.21 to 56.22%) and enzymes (34.3 to 40.76%) were major contributors to total raw material cost. Cost of ethanol production was observed to be sensitive to the pentose fermentation efficiency. Energy from lignin residue was sufficient to supply total steam required for ethanol production plant for all pretreatment processes. Energy use decreased and capital cost increased as the fraction of the liquid stream processed in evaporator decreased. Correspondingly, unit ethanol price increased.

## Competing interests

The authors declare that they have no competing interests.

## Authors' contributions

DK carried out the process simulations and wrote the paper. GM designed the study, helped in development of models and analyzed the results. GM reviewed the manuscript. All authors read and approved the final manuscript.

## Supplementary Material

Additional file 1 **Equipment cost and fixed capital estimate summary**. File contains eight tables. Four tables (A1, A3, A5 and A7) provide cost of major equipment used in different models. Other four tables (A2, A4, A6 and A8) provide summary of fixed capital cost for different models. Click here for file

Additional file 2 **SuperPro Designer Model for an ethanol production from grass straw using dilute acid pretreatment**. Detailed model for ethanol production plant using dilute acid pretreatment process. This model is for 250,000 metric ton grass straw (biomass) processing capacity. Click here for file

Additional file 3 **SuperPro Designer Model for an ethanol production from grass straw using dilute alkali pretreatment**. Detailed model for ethanol production plant using dilute alkali pretreatment. This model is for 250,000 metric ton grass straw (biomass) processing capacity. Click here for file

Additional file 4 **SuperPro Designer Model for an ethanol production from grass straw using hot water pretreatment**. Detailed model for ethanol production plant using hot water pretreatment. This model is for 250,000 metric ton grass straw (biomass) processing capacity. Click here for file

Additional file 5 **SuperPro Designer Model for an ethanol production from grass straw using steam explosion pretreatment**. Detailed model for ethanol production plant using steam explosion pretreatment. This model is for 250,000 metric ton grass straw (biomass) processing capacity. Click here for file
