# Supplementary material for: Impact of pretreatment and downstream processing technologies on economics and energy in cellulosic ethanol production
Source: Biotechnol Biofuels. 2011 Sep 5;4:27. doi: 10.1186/1754-6834-4-27 (PMC3179443; doi:10.1186/1754-6834-4-27)
Supplement: Additional file 1 — Equipment cost and fixed capital estimate summary. File contains eight tables. Four tables (A1, A3, A5 and A7) provide cost of major equipment used in different models. Other four tables (A2, A4, A6 and A8) provide summary of fixed capital cost for different models. [file 1754-6834-4-27-S1.PDF]

Appendix

Impact of pretreatment and downstream processing technologies on economics and energy use in cellulosic ethanol production

Deepak Kumar[1], Ganti S. Murthy[1*]

[1]Biological and Ecological Engineering, Oregon State University, Corvallis, USA

*Corresponding Author

Ganti S. Murthy

Assistant Professor

Biological and Ecological Engineering

116 Gilmore Hall, Oregon State University, Corvallis, OR-97331.

Ph. - 541-737-6291

murthy@engr.orst.edu

This Appendix of supporting information consists of 10 pages which include eight tables as follows:

Table A1 Major equipment specification and cost (2010 prices $) for ethanol production plant using dilute acid pretreatment process

Table A2 Fixed capital estimate summary (2010 prices $) for ethanol production plant using dilute acid pretreatment process

Table A3 Major equipment specification and cost (2010 prices $) for ethanol production plant using dilute alkali pretreatment process

Table A4 Fixed capital estimate summary (2010 prices $) for ethanol production plant using dilute alkali pretreatment process

Table A5 Major equipment specification and cost (2010 prices $) for ethanol production plant using hot water pretreatment process

Table A6 Fixed capital estimate summary (2010 prices $) for ethanol production plant using hot water pretreatment process

Table A7 Major equipment specification and cost (2010 prices $) for ethanol production plant using steam explosion pretreatment process

Table A8 Fixed capital estimate summary (2010 prices $) for ethanol production plant using steam explosion pretreatment process

Table A1 Major equipment specification and cost (2010 prices $) for ethanol production plant using dilute acid pretreatment process

Quantity/ Standby/ Staggered	Name	Description	Unit Cost ($)	Cost ($)
1 / 0 / 0	BC-101	Belt Conveyor	450000.00	450000.00
		Belt Length = 400.00 ft		
1 / 0 / 0	CSP-104	Component Splitter	3000.00	3000.00
		Size/Capacity = 97858.82 kg/h		
4 / 0 / 0	HX-101	Heat Exchanger	141000.00	564000.00
		Heat Exchange Area = 304.55 m2		
5 / 0 / 0	R-102	Fermentation Tank	773000.00	3865000.00
		Vessel Volume = 3904.13 m3		
1 / 0 / 0	V-105	DAP Storage Tank	18000.00	18000.00
		Vessel Volume = 1307.19 L		
1 / 0 / 0	V-107	Cellulase Storage Tank	115000.00	115000.00
		Vessel Volume = 69760.02 gal		
1 / 0 / 0	V-103	Yeast Storage Tank	22000.00	22000.00
		Vessel Volume = 4268.03 L		
1 / 0 / 0	V-104	Beer Well	178000.00	178000.00
		Vessel Volume = 168320.40 gal		
1 / 0 / 0	SR-101	Shredder	639000.00	639000.00
		Size/Capacity = 49749.38 kg/h		
1 / 0 / 0	C-102	Distillation Column	116000.00	116000.00
		Column Volume = 5.20 m3		
1 / 0 / 0	C-101	Distillation Column	177000.00	177000.00
		Column Volume = 2.87 m3		
1 / 0 / 0	CSP-101	Component Splitter	1155000.00	1155000.00
		Size/Capacity = 8236.95 kg/h		
1 / 0 / 0	HX-102	Heat Exchanger	8000.00	8000.00
		Heat Exchange Area = 6.48 m2		
1 / 0 / 0	PM-101	Centrifugal Pump	99000.00	99000.00
		Pump Power = 60.25 kW		
1 / 0 / 0	PM-102	Centrifugal Pump	38000.00	38000.00
		Pump Power = 6.12 kW		
2 / 0 / 0	HX-103	Heat Exchanger	141000.00	282000.00
		Heat Exchange Area = 370.78 m2		
1 / 0 / 0	HX-104	Heat Exchanger	76000.00	76000.00
		Heat Exchange Area = 106.58 m2		
1 / 0 / 0	EV-101	Evaporator	1609000.00	1609000.00
		Evaporation Area = 14154.29 ft2		
2 / 0 / 0	AD-101	Anaerobic Digester	1421000.00	2842000.00
		Vessel Volume = 914584.90 gal		
1 / 0 / 0	PFR-101	Pretreatment Reactor	4908000.00	4908000.00
1 / 0 / 0	AB-101	Aerobic Digester	53000.00	53000.00
		Vessel Volume = 4068.44 m3		
1 / 0 / 0	BF-101	Belt Filter	235000.00	235000.00
		Belt Width = 0.21 m		
1 / 0 / 0	HX-105	Heat Exchanger	54000.00	54000.00
		Heat Exchange Area = 19.22 m2		
1 / 0 / 0	GBX-105	Turbine/Generator	2729000.00	2729000.00

		Size/Capacity = 38243.50 kg/h		
1 / 0 / 0	GBX-101	Fluidized Bed Reactor	7312000.00	7312000.00
		Size/Capacity = 38243.50 kg/h		
1 / 0 / 0	HX-106	Heat Exchanger	7000.00	7000.00
		Heat Exchange Area = 15.82 m2		
1 / 0 / 0	V-102	Neutralizer	83000.00	83000.00
		Vessel Volume = 122.68 m3		
1 / 0 / 0	CY-101	Vacuum Filter and Hydrocyclone	199000.00	199000.00
		Rated Throughput = 111838.66 L/h		
2 / 0 / 0	GBX-104	Pneumapress	2291000.00	4582000.00
		Size/Capacity = 74252.04 kg/h		
1 / 0 / 0	V-101	Neutralizer	3000.00	3000.00
		Vessel Volume = 4133.26 L		
1 / 0 / 0	PM-103	Centrifugal Pump	12000.00	12000.00
		Pump Power = 0.46 kW		
2 / 0 / 0	GBX-106	Pneumapress Filter	1881000.00	3762000.00
		Size/Capacity = 63108.17 kg/h		
1 / 0 / 0	V-106	Sulfuric Acid Storage Tank	11000.00	11000.00
		Vessel Volume = 9153.81 gal		
1 / 0 / 0	V-108	Gasoline Storage	11000.00	11000.00
		Vessel Volume = 2762.51 gal		
1 / 0 / 0	HX-107	Heat Exchanger	11000.00	11000.00
		Heat Exchange Area = 33.33 m2		
1 / 0 / 0	HX-108	Heat Exchanger	7000.00	7000.00
		Heat Exchange Area = 15.21 m2		
1 / 0 / 0	PM-104	Centrifugal Pump	65000.00	65000.00
		Pump Power = 21.05 kW		
		Unlisted Equipment		9075000.00
			TOTAL	45373000

Table A2 Fixed capital estimate summary (2010 prices $) for ethanol production plant using dilute acid pretreatment process

A. Total Plant Direct Cost (TPDC) (physical cost)	
1. Equipment Purchase Cost	45373000.00
2. Installation	19182000.00
3. Process Piping	2269000.00
4. Instrumentation	2269000.00
5. Insulation	2269000.00
6. Electrical	2269000.00
7. Buildings	2269000.00
8. Yard Improvement	2269000.00
9. Auxiliary Facilities	2269000.00
TPDC	80436000.00
B. Total Plant Indirect Cost (TPIC)	
10. Engineering	4022000.00
11. Construction	8044000.00
TPIC	12065000.00

C. Total Plant Cost (TPC = TPDC+TPIC)	
TPC	92501000.00
D. Contractor's Fee & Contingency (CFC)	
12. Contractor's Fee	4625000.00
13. Contingency	9250000.00
CFC = 12+13	13875000.00
E. Direct Fixed Capital Cost (DFC = TPC+CFC)	
DFC	106376000

Table A3 Major equipment specification and cost (2010 prices $) for ethanol production plant using dilute alkali pretreatment process

Quantity/ Standby/ Staggered	Name	Description	Unit Cost ($)	Cost ($)
1 / 0 / 0	BC-101	Belt Conveyor	450000.00	450000.00
		Belt Length = 400.00 ft		
1 / 0 / 0	CSP-104	Component Splitter	3000.00	3000.00
		Size/Capacity = 97858.82 kg/h		
3 / 0 / 0	HX-101	Heat Exchanger	141000.00	423000.00
		Heat Exchange Area = 398.76 m2		
5 / 0 / 0	R-102	Fermentation Tank	773000.00	3865000.00
		Vessel Volume = 3901.21 m3		
1 / 0 / 0	V-105	DAP Storage Tank	18000.00	18000.00
		Vessel Volume = 1307.19 L		
1 / 0 / 0	V-107	Cellulase Storage Tank	129000.00	129000.00
		Vessel Volume = 80244.95 gal		
1 / 0 / 0	V-103	Yeast Storage Tank	22000.00	22000.00
		Vessel Volume = 4268.03 L		
1 / 0 / 0	V-104	Beer Well	177000.00	177000.00
		Vessel Volume = 168431.86 gal		
1 / 0 / 0	SR-101	Shredder	613000.00	613000.00
		Size/Capacity = 49749.38 kg/h		
1 / 0 / 0	C-102	Distillation Column	116000.00	116000.00
		Column Volume = 5.19 m3		
1 / 0 / 0	C-101	Distillation Column	177000.00	177000.00
		Column Volume = 2.86 m3		
1 / 0 / 0	CSP-101	Component Splitter	1152000.00	1152000.00
		Size/Capacity = 8212.57 kg/h		
1 / 0 / 0	HX-102	Heat Exchanger	9000.00	9000.00
		Heat Exchange Area = 6.81 m2		
1 / 0 / 0	PM-101	Centrifugal Pump	99000.00	99000.00
		Pump Power = 59.78 kW		
1 / 0 / 0	PM-102	Centrifugal Pump	38000.00	38000.00
		Pump Power = 6.12 kW		
2 / 0 / 0	HX-103	Heat Exchanger	141000.00	282000.00
		Heat Exchange Area = 354.60 m2		
1 / 0 / 0	HX-104	Heat Exchanger	91000.00	91000.00
		Heat Exchange Area = 132.58 m2		
1 / 0 / 0	EV-101	Evaporator	1580000.00	1580000.00

		Evaporation Area = 13778.09 ft2		
2 / 0 / 0	AD-101	Anaerobic Digester	1425000.00	2850000.00
		Vessel Volume = 3478.71 m3		
1 / 0 / 0	AB-101	Aerobic Digester	53000.00	53000.00
		Vessel Volume = 4088.43 m3		
1 / 0 / 0	BF-101	Belt Filter	235000.00	235000.00
		Belt Width = 0.19 m		
1 / 0 / 0	HX-105	Heat Exchanger	54000.00	54000.00
		Heat Exchange Area = 19.20 m2		
1 / 0 / 0	GBX-105	Turbine/Generator	2729000.00	2729000.00
		Size/Capacity = 40533.01 kg/h		
1 / 0 / 0	GBX-101	Fluidized Bed Reactor	7638000.00	7638000.00
		Size/Capacity = 40533.01 kg/h		
1 / 0 / 0	HX-106	Heat Exchanger	7000.00	7000.00
		Heat Exchange Area = 15.90 m2		
1 / 0 / 0	PM-103	Centrifugal Pump	12000.00	12000.00
		Pump Power = 0.46 kW		
2 / 0 / 0	GBX-106	Pneumapress Filter	1888000.00	3776000.00
		Size/Capacity = 63498.84 kg/h		
1 / 0 / 0	PFR-101	Pretreatment Reactor	4908000.00	4908000.00
1 / 0 / 0	V-101	Neutralizer	3000.00	3000.00
		Vessel Volume = 5371.38 L		
1 / 0 / 0	V-106	Alkali Storage Tank	11000.00	11000.00
		Vessel Volume = 8685.54 gal		
1 / 0 / 0	V-108	Gasoline Storage	11000.00	11000.00
		Vessel Volume = 2753.70 gal		
1 / 0 / 0	PM-104	Centrifugal Pump	65000.00	65000.00
		Pump Power = 21.06 kW		
1 / 0 / 0	HX-108	Heat Exchanger	7000.00	7000.00
		Heat Exchange Area = 15.17 m2		
1 / 0 / 0	HX-107	Heat Exchanger	11000.00	11000.00
		Heat Exchange Area = 33.06 m2		
		Unlisted Equipment		7903000.00
			TOTAL	39515000

Table A4 Fixed capital estimate summary (2010 prices $) for ethanol production plant using dilute alkali pretreatment process

A. Total Plant Direct Cost (TPDC) (physical cost)	
1. Equipment Purchase Cost	39515000.00
2. Installation	18247000.00
3. Process Piping	1976000.00
4. Instrumentation	1976000.00
5. Insulation	1976000.00
6. Electrical	1976000.00
7. Buildings	1976000.00
8. Yard Improvement	1976000.00
9. Auxiliary Facilities	1976000.00
TPDC	71592000.00

B. Total Plant Indirect Cost (TPIC)	
10. Engineering	3580000.00
11. Construction	7159000.00
TPIC	10739000.00
C. Total Plant Cost (TPC = TPDC+TPIC)	
TPC	82331000.00
D. Contractor's Fee & Contingency (CFC)	
12. Contractor's Fee	4117000.00
13. Contingency	8233000.00
CFC = 12+13	12350000.00
E. Direct Fixed Capital Cost (DFC = TPC+CFC)	
DFC	94680000

Table A5 Major equipment specification and cost (2010 prices $) for ethanol production plant using hot water pretreatment process

Quantity/ Standby/ Staggered	Name	Description	Unit Cost ($)	Cost ($)
1 / 0 / 0	BC-101	Belt Conveyor	450000.00	450000.00
		Belt Length = 400.00 ft		
1 / 0 / 0	CSP-104	Component Splitter	3000.00	3000.00
		Size/Capacity = 97858.82 kg/h		
2 / 0 / 0	HX-101	Heat Exchanger	141000.00	282000.00
		Heat Exchange Area = 345.78 m2		
5 / 0 / 0	R-102	Fermentation Tank	773000.00	3865000.00
		Vessel Volume = 3902.11 m3		
1 / 0 / 0	V-105	DAP Storage Tank	18000.00	18000.00
		Vessel Volume = 1307.19 L		
1 / 0 / 0	V-107	Cellulase Storage Tank	129000.00	129000.00
		Vessel Volume = 80132.28 gal		
1 / 0 / 0	V-103	Yeast Storage Tank	22000.00	22000.00
		Vessel Volume = 4268.03 L		
1 / 0 / 0	V-104	Beer Well	177000.00	177000.00
		Vessel Volume = 168168.97 gal		
1 / 0 / 0	SR-101	Shredder	613000.00	613000.00
		Size/Capacity = 49749.38 kg/h		
1 / 0 / 0	C-102	Distillation Column	116000.00	116000.00
		Column Volume = 5.20 m3		
1 / 0 / 0	C-101	Distillation Column	177000.00	177000.00
		Column Volume = 2.86 m3		
1 / 0 / 0	CSP-101	Component Splitter	1151000.00	1151000.00
		Size/Capacity = 8198.20 kg/h		
1 / 0 / 0	HX-102	Heat Exchanger	9000.00	9000.00
		Heat Exchange Area = 6.77 m2		
1 / 0 / 0	PM-101	Centrifugal Pump	99000.00	99000.00
		Pump Power = 59.73 kW		
1 / 0 / 0	PM-102	Centrifugal Pump	38000.00	38000.00

		Pump Power = 6.11 kW		
2 / 0 / 0	HX-103	Heat Exchanger	141000.00	282000.00
		Heat Exchange Area = 273.72 m2		
1 / 0 / 0	HX-104	Heat Exchanger	116000.00	116000.00
		Heat Exchange Area = 179.37 m2		
1 / 0 / 0	EV-101	Evaporator	1891000.00	1891000.00
		Evaporation Area = 17952.88 ft2		
2 / 0 / 0	AD-101	Anaerobic Digester	1420000.00	2840000.00
		Vessel Volume = 3455.69 m3		
1 / 0 / 0	AB-101	Aerobic Digester	53000.00	53000.00
		Vessel Volume = 4060.16 m3		
1 / 0 / 0	BF-101	Belt Filter	235000.00	235000.00
		Belt Width = 0.22 m		
1 / 0 / 0	HX-105	Heat Exchanger	54000.00	54000.00
		Heat Exchange Area = 19.25 m2		
1 / 0 / 0	GBX-105	Turbine/Generator	2729000.00	2729000.00
		Size/Capacity = 37546.12 kg/h		
1 / 0 / 0	GBX-101	Fluidized Bed Reactor	7212000.00	7212000.00
		Size/Capacity = 37546.12 kg/h		
1 / 0 / 0	HX-106	Heat Exchanger	7000.00	7000.00
		Heat Exchange Area = 15.82 m2		
1 / 0 / 0	PM-103	Centrifugal Pump	12000.00	12000.00
		Pump Power = 0.46 kW		
2 / 0 / 0	GBX-106	Pneumapress Filter	1878000.00	3756000.00
		Size/Capacity = 62961.32 kg/h		
1 / 0 / 0	PFR-101	Pretreatment Reactor	4908000.00	4908000.00
1 / 0 / 0	V-108	Gasoline Storage	11000.00	11000.00
		Vessel Volume = 2748.03 gal		
1 / 0 / 0	PM-104	Centrifugal Pump	65000.00	65000.00
		Pump Power = 21.03 kW		
1 / 0 / 0	HX-107	Heat Exchanger	11000.00	11000.00
		Heat Exchange Area = 33.13 m2		
1 / 0 / 0	HX-108	Heat Exchanger	7000.00	7000.00
		Heat Exchange Area = 15.14 m2		
		Unlisted Equipment		7834000.00
			TOTAL	39170000

Table A6 Fixed capital estimate summary (2010 prices $)for ethanol production plant using hot water pretreatment process

3A. Total Plant Direct Cost (TPDC) (physical cost)	
1. Equipment Purchase Cost	39170000.00
2. Installation	18376000.00
3. Process Piping	1959000.00
4. Instrumentation	1959000.00
5. Insulation	1959000.00
6. Electrical	1959000.00
7. Buildings	1959000.00
8. Yard Improvement	1959000.00

9. Auxiliary Facilities	1959000.00
TPDC	71256000.00
3B. Total Plant Indirect Cost (TPIC)	
10. Engineering	3563000.00
11. Construction	7126000.00
TPIC	10688000.00
3C. Total Plant Cost (TPC = TPDC+TPIC)	
TPC	81944000.00
3D. Contractor's Fee & Contingency (CFC)	
12. Contractor's Fee	4097000.00
13. Contingency	8194000.00
CFC = 12+13	12292000.00
3E. Direct Fixed Capital Cost (DFC = TPC+CFC)	
DFC	94236000

Table A7 Major equipment specification and cost (2010 prices $) for ethanol production plant using steam explosion pretreatment process

Quantity/ Standby/ Staggered	Name	Description	Unit Cost ($)	Cost ($)
1 / 0 / 0	BC-101	Belt Conveyor	450000.00	450000.00
		Belt Length = 400.00 ft		
1 / 0 / 0	CSP-104	Component Splitter	3000.00	3000.00
		Size/Capacity = 97858.82 kg/h		
1 / 0 / 0	HX-101	Heat Exchanger	141000.00	141000.00
		Heat Exchange Area = 48.52 m2		
4 / 0 / 0	R-102	Fermentation Tank	688000.00	2752000.00
		Vessel Volume = 850360.99 gal		
1 / 0 / 0	V-105	DAP Storage Tank	18000.00	18000.00
		Vessel Volume = 1307.19 L		
1 / 0 / 0	V-107	Cellulase Storage Tank	124000.00	124000.00
		Vessel Volume = 76454.42 gal		
1 / 0 / 0	V-103	Yeast Storage Tank	22000.00	22000.00
		Vessel Volume = 4268.03 L		
1 / 0 / 0	V-104	Beer Well	131000.00	131000.00
		Vessel Volume = 110115.69 gal		
1 / 0 / 0	SR-101	Shredder	613000.00	613000.00
		Size/Capacity = 49749.38 kg/h		
1 / 0 / 0	C-102	Distillation Column	89000.00	89000.00
		Column Volume = 3.33 m3		
1 / 0 / 0	C-101	Distillation Column	161000.00	161000.00
		Column Volume = 2.43 m3		
1 / 0 / 0	CSP-101	Component Splitter	1064000.00	1064000.00
		Size/Capacity = 7187.07 kg/h		
1 / 0 / 0	HX-102	Heat Exchanger	8000.00	8000.00
		Heat Exchange Area = 6.21 m2		
1 / 0 / 0	PM-101	Centrifugal Pump	83000.00	83000.00

		Pump Power = 38.90 kW		
1 / 0 / 0	PM-102	Centrifugal Pump	31000.00	31000.00
		Pump Power = 3.95 kW		
2 / 0 / 0	HX-103	Heat Exchanger	141000.00	282000.00
		Heat Exchange Area = 206.42 m2		
1 / 0 / 0	HX-104	Heat Exchanger	64000.00	64000.00
		Heat Exchange Area = 85.41 m2		
1 / 0 / 0	EV-101	Evaporator	1193000.00	1193000.00
		Evaporation Area = 9116.26 ft2		
1 / 0 / 0	AD-101	Anaerobic Digester	1575000.00	1575000.00
		Vessel Volume = 4236.35 m3		
1 / 0 / 0	PFR-101	Pretreatment Reactor	3896000.00	3896000.00
1 / 0 / 0	AB-101	Aerobic Digester	32000.00	32000.00
		Vessel Volume = 2463.54 m3		
1 / 0 / 0	BF-101	Belt Filter	235000.00	235000.00
		Belt Width = 0.22 m		
1 / 0 / 0	HX-105	Heat Exchanger	54000.00	54000.00
		Heat Exchange Area = 10.97 m2		
1 / 0 / 0	GBX-105	Turbine/Generator	4421000.00	4421000.00
		Size/Capacity = 34175.87 kg/h		
1 / 0 / 0	GBX-101	Fluidized Bed Reactor	6721000.00	6721000.00
		Size/Capacity = 34175.87 kg/h		
1 / 0 / 0	HX-106	Heat Exchanger	5000.00	5000.00
		Heat Exchange Area = 11.41 m2		
1 / 0 / 0	V-101	Flash Drum	31000.00	31000.00
		Vessel Volume = 1117.71 gal		
1 / 0 / 0	PM-103	Centrifugal Pump	10000.00	10000.00
		Pump Power = 0.27 kW		
1 / 0 / 0	G-101	Centrifugal Compressor	625000.00	625000.00
		Compressor Power = 548.65 kW		
1 / 0 / 0	HX-107	Heat Exchanger	141000.00	141000.00
		Heat Exchange Area = 214.57 m2		
2 / 0 / 0	GBX-106	Pneumapress Filter	1456000.00	2912000.00
		Size/Capacity = 41200.26 kg/h		
1 / 0 / 0	V-102	Gasoline Storage	10000.00	10000.00
		Vessel Volume = 2476.53 gal		
1 / 0 / 0	HX-108	Heat Exchanger	13000.00	13000.00
		Heat Exchange Area = 41.11 m2		
1 / 0 / 0	PM-104	Centrifugal Pump	55000.00	55000.00
		Pump Power = 13.77 kW		
1 / 0 / 0	HX-109	Heat Exchanger	6000.00	6000.00
		Heat Exchange Area = 13.62 m2		
		Unlisted Equipment		6993000.00
			TOTAL	34963000

Table A8 Fixed capital estimate summary (2010 prices $) for ethanol production plant using steam explosion pretreatment process

3A. Total Plant Direct Cost (TPDC) (physical cost)

1. Equipment Purchase Cost	34963000.00
2. Installation	16512000.00
3. Process Piping	1748000.00
4. Instrumentation	1748000.00
5. Insulation	1748000.00
6. Electrical	1748000.00
7. Buildings	1748000.00
8. Yard Improvement	1748000.00
9. Auxiliary Facilities	1748000.00
TPDC	63712000.00
3B. Total Plant Indirect Cost (TPIC)	
10. Engineering	3186000.00
11. Construction	6371000.00
TPIC	9557000.00
3C. Total Plant Cost (TPC = TPDC+TPIC)	
TPC	73269000.00
3D. Contractor's Fee & Contingency (CFC)	
12. Contractor's Fee	3663000.00
13. Contingency	7327000.00
CFC = 12+13	10990000.00
3E. Direct Fixed Capital Cost (DFC = TPC+CFC)	
DFC	84259000
